# A novel prediction model for pathological complete response based on clinical and blood parameters in locally advanced rectal cancer

**DOI:** 10.3389/fonc.2022.932853

**Published:** 2022-11-23

**Authors:** Siyi Lu, Zhenzhen Liu, Yuxia Wang, Yan Meng, Ran Peng, Ruize Qu, Zhipeng Zhang, Wei Fu, Hao Wang

**Affiliations:** ^1^ Department of General Surgery, Peking University Third Hospital, Beijing, China; ^2^ Department of Thoracic Surgery, Beijing Jishuitan Hospital, Beijing, China; ^3^ Department of Radiation Oncology, Peking University Third Hospital, Beijing, China; ^4^ Cancer Center, Peking University Third Hospital, Beijing, China

**Keywords:** pathological complete response, locally advanced rectal cancer, prediction model, neoadjuvant chemotherapy, nomogram

## Abstract

**Background:**

The aim of this study was to investigate whether clinical and blood parameters can be used for predicting pathological complete response (pCR) to neoadjuvant chemoradiotherapy (nCRT) in patients with locally advanced rectal cancer (LARC).

**Methods:**

We retrospectively enrolled 226 patients with LARC [allocated in a 7:3 ratio to a training (n = 158) or validation (n = 68) cohort] who received nCRT before radical surgery. Backward stepwise logistic regression was performed to identify clinical and blood parameters associated with achieving pCR. Models based on clinical parameters (CP), blood parameters (BP), and clinical-blood parameters (CBP) were constructed for comparison with previously reported Tan’s model. The performance of the four models was evaluated by receiver operating characteristic (ROC) curve analysis, calibration, and decision curve analysis (DCA) in both cohorts. A dynamic nomogram was constructed for the presentation of the best model.

**Results:**

The CP and BP models based on multivariate logistic regression analysis showed that interval, Grade, CEA and fibrinogen–albumin ratio index (FARI), sodium-to-globulin ratio (SGR) were the independent clinical and blood predictors for achieving pCR, respectively. The area under the ROC curve of the CBP model achieved a score of 0.818 and 0.752 in both cohorts, better than CP (0.762 and 0.589), BP (0.695 and 0.718), Tan (0.738 and 0.552). CBP also showed better calibration and DCA than other models in both cohorts. Moreover, CBP revealed significant improvement compared with other models in training cohort (*P* < 0.05), and CBP showed significant improvement compared with CP and Tan’s model in validation cohort (*P* < 0.05).

**Conclusion:**

We demonstrated that CBP predicting model have potential in predicting pCR to nCRT in patient with LARC.

## Background

Neoadjuvant chemoradiotherapy (nCRT) followed by total mesorectal excision (TME) has become the conventional care for locally advanced rectal cancer (LARC) (1). After nCRT, about 15% to 27% of patients respond well to radiotherapy and chemotherapy, will achieve a pathological complete response (pCR), in which no tumor is found in the surgical resection specimen after nCRT (2, 3). Based on this, recent studies recommend a “watch and wait” management can be adopted for patients who judged to be complete clinical response (cCR) after nCRT, that is, only close follow-up is performed without surgical intervention, and salvage surgery can be considered when tumor recurrence occurs (4, 5), so as to achieve the purpose of organ preservation and protect patients from surgery-associated morbidity and the associated impairment in quality of life (6, 7).

However, the difficulty in the promotion of this concept lies in the accurate determination of tumor response to nCRT (8). Although there are different clinical methods, including digital rectal examination, tumor marker examination, imaging and preoperative colonoscopy, have been used to evaluate clinically tumor response after nCRT, their sensitivity and specificity are unsatisfactory, and the coincidence rate between cCR and postoperative pathological assessment was still less than 50% (9–11). It turns out that single-predictor might not be a proper strategy for pCR prediction. Therefore, it is an urgent clinical problem to explore the reliable parameters and construct predicting models for predicting pCR after nCRT for LARC.

Many researchers have conducted research on tumor response of LARC and found that predicting models based on clinical parameters and/or blood parameters are both valuable for predicting pCR (12–15). In a recent large large-sample study based on Surveillance Epidemiology and End Results (SEER) database, Tan et al. constructed a nomogram comprised several clinical parameters to predict pCR, and the nomogram model which including histology, Grade, CEA, cT stage, and cN stage, shown a great practical value for predicting pCR (13). In addition, some researchers also developed new models for predicting treatment response in a variety of tumor types based on blood test parameters, such as fibrinogen-to-albumin ratio index (FARI) (16) and sodium-to-globulin ratio (SGR) (17), which also shown potential clinical value for treatment response interpretation. Our previous studies also showed that clinical and blood parameters were closely relative to the tumor response to nCRT (18, 19). We wonder whether integrating multiple parameters could obtain a robust model for predicting pCR.

Hence, in this study we aimed to develop, internal validate, and assess the performance of models to predict pCR status. In addition, a comparison between the constructed models and published model was conducted to determine which model provided more accurate prediction in pCR. After that, the best model will be visualized by dynamic nomogram in order to facilitate clinical application.

## Methods

### Study population

**Figure 1:**
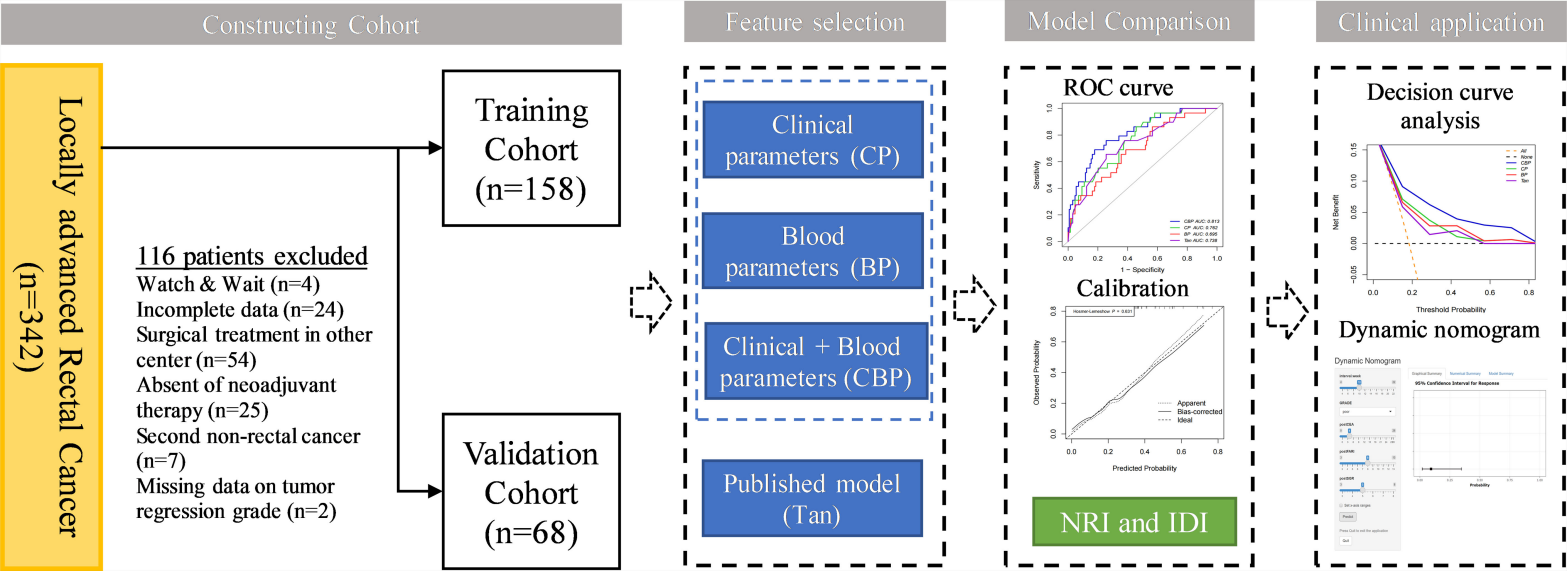
Flow chart of the study.

This retrospective single-center study was approved by Peking University Third Hospital (IRB00006761-M2019387), and this study adhered to the tenets of the Declaration of Helsinki. The requirement for informed consent was waived by the Institutional Review Board of Peking University Third Hospital. A total of 226 patients treated with nCRT between January 2012 and June 2021 at the Department of General Surgery at Peking University Third Hospital were retrospectively recruited. The inclusion criteria were as follows: 1) All patients underwent pretreatment colonoscopy biopsy confirming the diagnosis of adenocarcinoma or mucinous adenocarcinoma; 2) All patients diagnosis of LARC (cT3-4/N0-2/M0) through pretreatment CT and MRI; 3) All patients underwent nCRT followed by radical surgery; and 4) Pathological response was confirmed by experienced pathologists using AJCC TRG system. The exclusion criteria were as follows: 1) Management by a watch & wait strategy after nCRT; 2) Incomplete clinical information; 3) Tumor regression grade data of patient were unavailable; and 4) Patient with second primary cancer, autoimmune, hematological disease, acute or chronic infection. Patients were allocated to training and validation cohorts according to TRG status in a 7:3 ratio, the training cohort consisted of 158 patients, the validation cohort of 68 patients. The data analysis flowchart of the study is shown in **Figure 1**.

### Neoadjuvant chemoradiotherapy treatment and pathologic assessment of response

All patients were treated with the same nCRT treatment scheme. The decision to administer nCRT or conduct radical resection was made by a multidisciplinary team, which consisted of surgeons, oncologists, pathologists, and radiologists. All patients received a total radiation doses ranged from 45 to 50 Gy applied in 25 fractions, at 1.8 to 2.0 Gy per fraction delivered once a day. Radiation was given according to institutional protocols. The oral capecitabine dosage during the whole course of radiotherapy (RT) was 825 mg/m^2^ twice daily. Surgically resected specimens were histopathologically examined and analyzed by an experienced pathologist. The AJCC tumor regression grade (TRG) definitions were as follows: TRG0, no residual of tumor cells; TRG1, single tumor cell or small groups of tumor cells; TRG2, residual cancer with a desmoplastic response; and TRG3, no tumor cells killed (20).According to AJCC TRG system, patients were divided into two groups: pCR (TRG0) and non-pCR (TRG1-3).

### Clinical and blood parameters

Baseline clinical and blood parameters were collected retrospectively from the electronic medical record system in our institution. Clinical T stage and N stage were determined by multidisciplinary team before nCRT. Clinical parameters including basic clinical data, pathological information, clinical stage, carcinoembryonic antigen (CEA) level and carbohydrate antigen 19–9 (CA199) level. All patients underwent general preoperative blood tests including routine blood examinations, biochemical tests and tumor markers according to the standard treatment procedure within 2 weeks before surgery. Blood specimens were collected between 5:30 and 6:30 a.m. and tested in the laboratory of our hospital. Blood parameters were defined as follows: NLR (neutrophil-to-lymphocyte ratio) = (neutrophil count (10^9^/L)/lymphocyte count (10^9^/L)); LMR (lymphocyte-to-monocyte ratio) = (lymphocyte count (10^9^/L)/monocyte count (10^9^/L)); PLR (platelet-to-lymphocyte ratio) = (platelet count (10^9^/L)/lymphocyte count (10^9^/L)); SII (systematic immune-inflammation index) = (platelet count (10^9^/L)) × NLR; FARI = (fibrinogen (g/L)/albumin (g/L)) × 100%; PNI (prognostic nutrition index) = (albumin (g/L) + 5 × lymphocyte count (10^9^/L)); SGR = (sodium count (mmol/L)/globulin count (g/L)).

### Construction and comparison of different prediction models

To assess the risk factors for pCR in clinical and blood parameters, univariable analysis was performed in the training cohort. Potential risk factors (p < 0.2) of clinical and blood parameters were separately adopted for the multivariate analysis with the backward stepwise method, following the results of the univariate analysis. Clinical parameters (CP) and blood parameters (BP) were constructed as described above. We then combine the risk factors for pCR in clinical and blood parameters to construct the clinical-blood parameters (CBP) model. The model developed by Tan et al., which including histology, Grade, CEA, cT stage, and cN stage, was also included in our study for comparison (13).

Receiver operating characteristic (ROC) curve analysis was conducted in both cohorts to evaluate the predictive ability of different models. Delong test was applied to assess whether the significant of difference in area under the ROC curve (AUC) estimates between the models. Cut-off value of different models were determined by Youden index.

Calibration curves were plotted to assess the consistency between the estimated probability and the actual rate of pCR. Model goodness of fit was assessed using the Hosmer-Lemeshow goodness of fit test and *P*-value > 0.05 indicate a good fit of the model.

To evaluate reclassification (the ability of a new model to improve on a previous model) we calculated the categorical net reclassification improvement (NRI) and the integrated discrimination improvement (IDI) (21).

The clinical usefulness of different models was assessed *via* decision curve analysis (DCA), which present net clinical benefit against different decision thresholds for predicting pCR and are used to evaluate the utility of decisions made based on the different models (22).

### Statistical analysis

Continuous variables were expressed as mean ± standard deviation (SD) for normally distributed variables, and non-normally distributed variables were express as the median and the 25th and 75th quartile. Continuous variables were compared by means of Student’s t-test or the Wilcoxon rank sum test for continuous variables and chi-square test or Fisher’s exact test for categorical variables. ROC curve and Delong test were performed using the “pPCR” package, the calibration plot was examined through the “rms” package, DCA was constructed by the function “dca.R”, the NRI and IDI were calculated using the “PredictABEL” package, and the dynamic nomogram was built by the “DynNom” package. All statistical analyses were conducted using SPSS Statistics 24.0 (IBM Corporation, Armonk, NY, USA), R version 4.1.1 and GraphPad Prism version 9.0. A *P*-value of < 0.05 was recognized as statistically significant.

## Results

### Clinical characteristics

According to the inclusion and exclusion criteria, a total of 226 LARC patients were eventually enrolled in the study. Clinical characteristics of patients in different cohorts are summarized in **Table 1**. A total of 45 (19.9%) patients reached pCR after nCRT, which were randomly distributed in the training and validation cohorts, 29 (18.4%) and 16 (23.5%), respectively. There were no significant differences between the 2 cohorts in terms of pCR prevalence (*P* = 0.477), and there were no significant differences in other clinical and blood parameters between the training and validation cohorts except for distance to the anal verge (DTAV) and PNI (**Table 1**). However, both these two variables were not included in the models in subsequent study.

**Table 1:** Clinical characteristics of patients in the training and validation cohorts.

Characteristic	Training Cohort	Validation Cohort	*P*-value
**n**	158	68	
**Age, median (IQR)**	61 (53.25, 69)	60.5 (54, 67.25)	0.992
**Gender, n (%)**			0.519
Male	113 (71.5%)	45 (66.2%)	
Female	45 (28.5%)	23 (33.8%)	
**BMI, mean ± SD**	24.08 ± 3.11	24.23 ± 3.06	0.736
**Interval, median (IQR)**	10 (9, 11)	10 (8, 11)	0.143
**DTAV, median (IQR)**	5.55 (4, 7)	6.75 (5, 8.2)	0.024
**Tumor size, median (IQR)**	5 (4, 6)	5 (4.5, 5.8)	0.786
**Histology, n (%)**			1.000
Adenocarcinoma	141 (89.2%)	61 (89.7%)	
Mucinous	17 (10.8%)	7 (10.3%)	
**cT stage, n (%)**			0.252
cT2	9 (5.7%)	2 (2.9%)	
cT3	125 (79.1%)	50 (73.5%)	
cT4	24 (15.2%)	16 (23.5%)	
**cN status, n (%)**			1.000
Negative	39 (24.7%)	17 (25%)	
Positive	119 (75.3%)	51 (75%)	
**Grade, n (%)**			0.573
poor differentiation	17 (10.8%)	9 (13.2%)	
moderate differentiation	130 (82.3%)	52 (76.5%)	
well differentiation	11 (7%)	7 (10.3%)	
**TGR, n (%)**			0.477
Non-pCR	129 (81.6%)	52 (76.5%)	
pCR	29 (18.4%)	16 (23.5%)	
**CEA, median (IQR)**	2.64 (1.87, 4.45)	2.56 (1.75, 3.8)	0.199
**CA199, median (IQR)**	11.96 (6.95, 17.86)	10.23 (8.13, 16.64)	0.649
**NLR, median (IQR)**	4.19 (3.02, 5.31)	4.21 (3.54, 5.96)	0.271
**LMR, median (IQR)**	2.09 (1.55, 2.79)	2.08 (1.63, 2.55)	0.479
**PLR, median (IQR)**	259.36 (200.42, 321.3)	281.85 (191.66, 328.16)	0.363
**SII, median (IQR)**	773.53 (564.76, 1071.68)	795.55 (587.9, 1210.87)	0.419
**FARI, median (IQR)**	7.46 (6.63, 8.77)	7.62 (6.88, 9.24)	0.284
**PNI, mean ± SD**	45.66 ± 4.31	44.46 ± 3.59	0.033
**SGR, mean ± SD**	5.43 ± 0.78	5.27 ± 0.73	0.156

IQR, interquartile range; BMI, body mass index; SD, standard deviation; DTAV, distance to anal verge; TRG, tumor regression grade; pCR, pathological complete response; NLR, neutrophil-to-lymphocyte ratio; LMR, lymphocyte-to-monocyte ratio; PLR, platelet-to-lymphocyte ratio; SII, systemic immune–inflammation index; FARI, fibrinogen–to-albumin ratio index; PNI, prognostic nutritional index; SGR, sodium-to-globulin ratio.

P-values were calculated by Student’s t-test, Wilcoxon rank sum test, Chi-square test or Fisher exact test.

### Feature selection and predicting model construction

**Figure 2:**
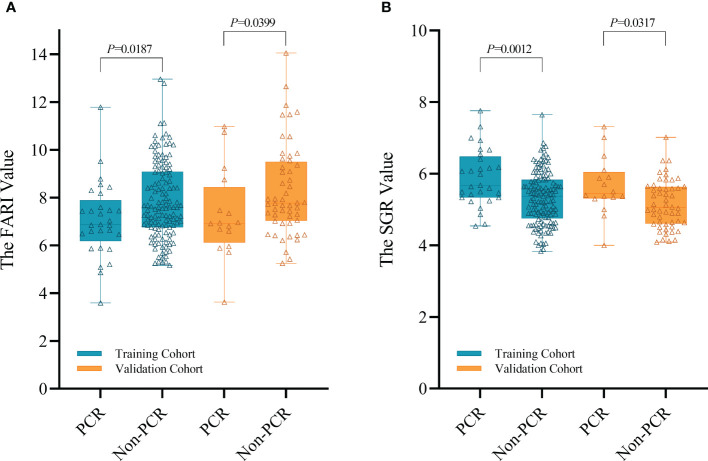
Distribution of the FARI **(A)** and SGR **(B)** in both cohorts.

Based on univariable and multivariable modelling of clinical parameters in the training cohort, we identified interval (interval weeks between end of nCRT and surgery) (OR 1.305, *P* = 0.01), Grade (*P* = 0.02) and CEA level (OR 0.743, *P* = 0.046) as independent risk factors for pCR (**Table 2**). CP model was then constructed based on these 3 variables. In training and validation cohorts, the FARI level and SGR level were significantly differences in the pCR group than in the non-pCR group, as shown in **Figure 2**. In addition, multivariate analysis of blood parameters in the training cohort was indicated that both FARI (OR 0.718, *P* = 0.036) and SGR (OR 2.555, *P* = 0.002) were significantly associated with pCR, as shown in **Table 3**. Model based on FARI and SGR was called BP model. Next, we integrated these clinical and blood parameters that significant significantly associated with pCR, and create a novel prediction model named CBP.

**Table 2:** Univariate and multivariate logistic regression analyses for clinical parameters.

Characteristics	pCR			
	Univariable		multivariable	
	OR (95%CI)	*P*-value	OR (95%CI)	*P*-value
**Age** (years)	0.978 (0.946-1.010)	0.179	–	–
**Gender** (male vs female)	0.947 (0.385-2.328)	0.906	–	–
**BMI** (kg/m^2^)	1.013 (0.890-1.154)	0.842	–	–
**Interval** (weeks)	1.224 (1.028-1.457)	0.023	1.305 (1.067-1.597)	0.010
**DTAV** (cm)	1.037 (0.896-1.201)	0.624	–	–
**Tumor size** (cm)	0.971 (0.763-1.236)	0.813	–	–
**Histology**	–	–	–	–
(Adenocarcinoma vs Mucinous)	0.252 (0.032-1.983)	0.190	–	–
**cT stage**	–	0.169	–	–
cT4 vs cT2	2.875 (0.160-51.534)	0.473	–	–
cT4 vs cT3	6.337 (0.818-49.073)	0.077	–	–
**cN status** (N+ vs N-)	1.318 (0.494-3.517)	0.582	–	–
**Grade**	–	0.015	–	0.020
Well vs Moderate	0.179 (0.032-0.999)	0.050	0.202 (0.033-1.239)	0.084
Well vs Poor	0.152 (0.042-0.544)	0.004	0.139 (0.035-0.555)	0.005
**CEA** (ng/ml)	0.731 (0.556-0.962)	0.025	0.743 (0.555-0.995)	0.046
**CA199** (kU/L)	0.988 (0.949-1.029)	0.560	–	–

pCR, pathological complete response; OR, odds ratio; CI, cofidence interval; BMI, body mass index; DTAV, distance to anal verge.

**Table 3:** Univariate and multivariate logistic regression analyses for blood parameters.

Characteristics	pCR			
	Univariable		multivariable	
	OR (95%CI)	*P*-value	OR (95%CI)	*P*-value
**NLR**	0.861 (0.695-1.067)	0.172	–	–
**LMR**	1.294 (0.870-1.925)	0.203	–	–
**PLR**	0.999 (0.996-1.002)	0.528	–	–
**SII**	1.000 (0.999-1.000)	0.270	–	–
**FARI**	0.707 (0.529-0.944)	0.019	0.718 (0.527-0.978)	0.036
**PNI**	1.057 (0.961-1.163)	0.254	–	–
**SGR**	2.626 (1.494-4.616)	0.001	2.555 (1.430-4.565)	0.002

pCR, pathological complete response; OR, odds ratio; CI, cofidence interval; NLR, neutrophil-to-lymphocyte ratio; LMR, lymphocyte-to-monocyte ratio; PLR, platelet-to-lymphocyte ratio; SII, systemic immune–inflammation index; FARI, fibrinogen–albumin ratio index; PNI, prognostic nutritional index; SGR, sodium-to-globulin ratio.

### Evaluation and comparison of different prediction models

**Figure 3:**
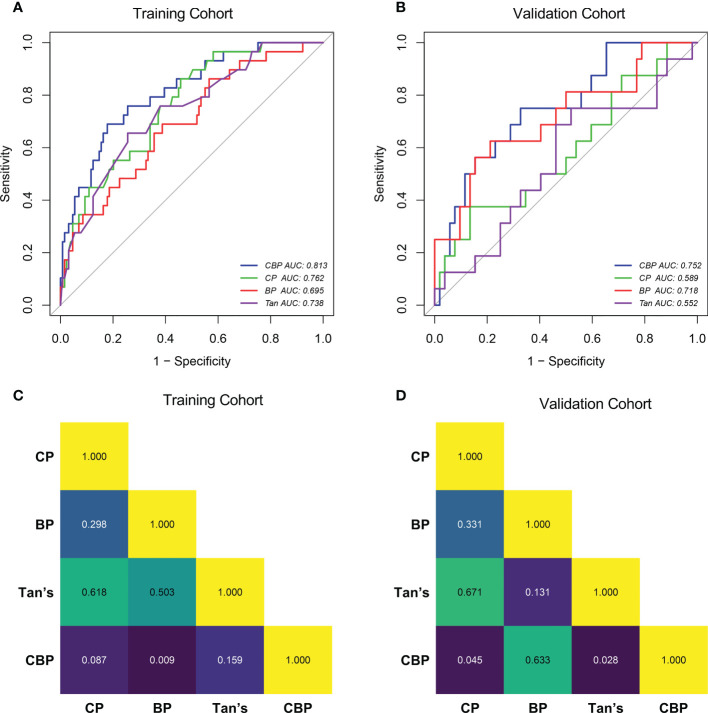
ROC curves of the CP, BP, CBP and Tan in Training cohort **(A)** and Validation cohort **(B)**. Delong test *P*-value of Training cohort **(C)** and Validation cohort **(D)**. CP clinical parameters model, BP blood parameters model, CBP clinical-blood parameters model.

Compared with other models, CBP model yielded the highest AUC in both cohorts (AUC = 0.813 in training cohort, AUC = 0.752 in validation cohort), and was statistically significantly higher than BP model (Delong test *P*-value = 0.009) in training cohort, CP model (Delong test *P*-value = 0.045) and Tan model (Delong test *P*-value = 0.028) in the validation cohort, as shown in **Figure 3**. The AUC, accuracy, sensitivity, and specificity of CP, BP, CBP and Tan models, according to the Youden index cut-off, are listed in **Supplementary Table 1**.

Compared with other models. the calibration curve generated from the CBP model presents a good consistency between the predicted risk and actual observation in both cohorts (Hosmer-Lemeshow test *P*-value > 0.05), as shown in **Figure S1**. Hosmer-Lemeshow tests indicated that other models fitted the data well (*P*-value > 0.05), except for Tan model’s goodness of fit in the validation cohort (*P*-value < 0.05).

After calibration, the calculated NRI and IDI further proved that the pCR predictive performance of CBP was better than CP, BP, and Tan. According to the quantitative results, the CBP model improved the predictive performance more significantly in the training cohort (*P* < 0.05), detail results are shown in **Table 4**. In validation cohort, CBP also present a better performance in prediction of pCR than CP (NRI and IDI *P*-value < 0.05) and Tan (IDI *P*-value < 0.05) model (**Table 4**).

**Table 4:** Model performance in the training and validation cohorts.

Model	Training Cohort			Validation Cohort		
	NRI	95%CI	*P-*value	NRI	95%CI	*P-*value
CBP vs. CP	0.207	0.029-0.385	0.023*	0.303	0.020-0.585	0.036*
CBP vs. BP	0.226	0.038-0.414	0.018*	0.106	-0.084-0.296	0.276
CBP vs. Tan	0.268	0.076-0.460	0.006*	0.313	-0.035-0.660	0.078
	**IDI**	**95%CI**	***P-*value**	**IDI**	**95%CI**	***P-*value**
CBP vs. CP	0.116	0.042-0.189	0.002*	0.074	0.005-0.147	0.048*
CBP vs. BP	0.141	0.073-0.210	<0.001*	0.045	-0.078-0.770	0.430
CBP vs. Tan	0.128	0.033-0.223	0.008*	0.113	0.003-0.223	0.045*

NRI, net reclassification improvement; CI, confidence interval; IDI, integrated discrimination improvement. CP, clinical parameters model; BP, blood parameters model; CBP, clinical-blood parameters model.

P-value <0.05, P-value were calculated by NRI test and IDI test.

### Clinical usefulness of different models and dynamic nomogram

**Figure 4:**
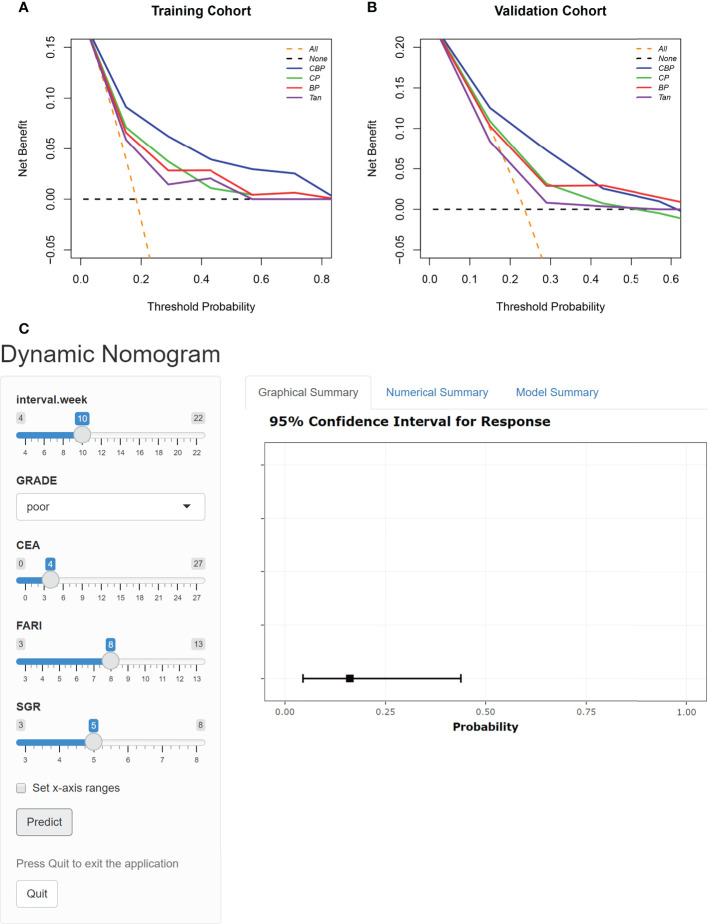
DCA for the four models in Training cohort **(A)** and Validation cohort **(B)**. The orange line represents the assumption that all patients showed PCR. The black line represents the assumption that no patients showed pCR. **(C)** Dynamic nomogram of CBP.

The DCA results of the different models in both cohorts are shown in **Figures 4A, B**. Using the proposed four models to detect pCR in both cohorts show a greater advantage than either the scheme in which all patients are assumed to achieve pCR or the scheme in which no patients are. The decision curve of CBP model was continuously superior to that of CP, BP, and Tan model in terms of clinical application in the training cohort. Similarly, the decision curve of CBP was higher than that of CP, BP, and Tan model in the probability of achieving pCR ranges from 0% to 40%. For wider and easier use of CBP model by clinicians and researchers, a dynamic nomogram was generated, as shown in **Figure 4C**. The nomograms of CP, BP, and Tan model were shown in **Supplementary Figure 2**.

## Discussion

In the present study, the accuracy of clinical parameters along (CP), blood parameters along (BP), and combining both clinical and blood parameters (CBP), and published model (Tan) for noninvasive, individualized prediction of pCR in patients with LARC undergoing nCRT was compared. The proposed CBP model performs best compared with other models and thereby provides an effective tool for clinical decision making. The easy-to-use dynamic nomogram facilitated noninvasive estimation of pCR.

“Watch and Wait” was recommended as an objective strategy for LARC patients. How long to be waited between completion of radiation and achievement of cCR, it varied from different research institute. Habr-Gama et al. proposed that intervals between completion of radiation and achievement of cCR may beyond 16 weeks in most cases (23). Similarly, a randomized trial of 252 LARC patients reported that the pCR rate of the 12-week interval group (23.6%) was significantly higher than the 8-week interval group (12%) (24). The multivariate analysis outcomes of interval (OR 1.305, *P* = 0.01) in this study was consistent with these previous studies, indicated that despite frequent early responses and reduction in tumor burden, a complete response of tumor may take longer than we think.

There is growing evidence that several clinical parameters play a critical role in the treatment response of LARC (25). Despite “watch and wait” strategy have been observed benefit in favor of patients with LARC from organ preservation, surgery-related morbidity, and quality of life (6, 7). It is difficult to diagnose the primary tumor to be a cCR status based on the common methods in clinical practice, and further to predict pCR (9–11). A recent research showed that tumor differentiation grade presents the same predictive role for pCR (26). However, Ono et al. did not observe such associations (27). We found that tumor differentiation grade (P = 0.02) was an independent risk factor for pCR in our study. This discrepancy could be due to a small sample size or the different nCRT regimens. Previous studies showed that compared with the pre-nCRT CEA level, both the post-nCRT CEA level and the change pattern of CEA during nCRT, are closely related to nCRT response of LARC patients (28, 29). We found post-nCRT CEA level (OR 0.743, P = 0.046) was an another potentially important predictor of pCR. This might due to the latter values reflect the degree of nCRT response. This suggested that the performance of a predictive model would be enhanced using data obtained during or after CRT.

Recently, various blood parameters, which reflecting systematic inflammatory response and nutrition status, seem to affect the tumor response to nCRT in patients with malignancy (14, 18, 19, 30). Of note, numerous studies have demonstrated that the systematic inflammatory response and nutrition status could destroy immune systems and increase tumor resistance to nCRT (31, 32). In multivariate analysis, other than FARI (OR 0.718, *P* = 0.036) and SGR (OR 2.555, *P* = 0.002), both NLR, LMR, PLR, SII, and PNI are failed to predict pCR. A potential reason for this inconsistent result is that except from FARI and SGR, these blood parameters were all leukocyte-based markers. However, in our study cohorts, all patients received nCRT, which would cause the bone marrow suppression. This may lead to these leukocyte-based markers fail to reflect the real systemic inflammatory response of patients, lowering the accuracy of these markers in predicting pCR. This is in line with the findings of Wang et al. (33) and our previous study (18).

Since the poor performance of singe-predictor to predicting pCR (9–11), predicting models integrating multiple parameters are receiving more and more attention in the field of rectal cancer as increasing evidence is gained about their promising performance in predicting pCR. Recent studies from Ren et al. (12) and Zhang et al. (34) found that the pCR predicting models comprised with clinical parameters and nCRT regimens performed exceedingly well, and the C-index were 0.793 and 0.802, respectively. However, the patients in this study were underwent the same nCRT regimen so it was impossible to explore its effect on pCR. In addition to the clinical based models, predicting models combined with blood parameters were also showed good performance in pCR prediction. A pCR predicting model based on NLR, LMR, and neutrophil-monocyte to lymphocyte ratio (NMLR) achieving an AUC of 0.75 (35). Though promising, these published models have been limited by the lack of an independent validation cohort. Besides, more detail information of these models, such as sensitivity, specificity, and accuracy have not clarified. Considering different tumor behaviors are determined by many interactions among multiple factors. Hence, in the current study, a CBP model combined with clinical and blood parameters was built. Among the four models, only CBP model (AUC = 0.816 in training cohort and AUC = 0.752 in validation cohort) achieved an AUC > 0.75, indicating acceptable prediction. The result of calibration and DCA further confirmed our previous conjectures. Moreover, when comparing CBP model with the CP, BP, and Tan model through NRI and IDI, the improvement in prediction accuracy was all significant in training cohort (all *P* < 0.05, NRI test, IDI test), and CBP model showed significant improvement compared with CP (*P* < 0.05, NRI test, IDI test) and Tan model in validation cohort (*P* < 0.05, IDI test).

Finally, the developed dynamic nomogram allows the CBP model to be used conveniently in clinical practice.

The present study has limitations. First, our study is limited by its retrospective design, selection bias cannot be ruled out. Second, because of the small sample size, randomization resulted in imbalance of DTAV and PNI between training and validation cohort. Additionally, possibly for the same reason, some NRI and IDI results in validation cohort showed a consistent tend with the training cohort, but the difference did not reach statistical significance. Finally, although we categorized the patients into independent training and validation cohorts according to their TRG status, the CBP model may therefore perform less well in other situations because of the lack of an external validation. A multicenter approach would have given more external validity to our estimates.

## Conclusion

In summary, we developed an easy-to-use and validated model, which combined with clinical parameters and blood parameters, can allow more accurate prediction of pCR in patients with LARC. This noninvasive and convenient CBP model may offer a novel way to the detection of pCR and assist clinicians in clinical decision-making, potentially.

## Data availability statement

The raw data supporting the conclusions of this article will be made available by the authors, without undue reservation.

## Ethics statement

This retrospective single-center study was approved by Peking University Third Hospital (IRB00006761-M2019387), and this study adhered to the tenets of the Declaration of Helsinki. The requirement for informed consent was waived by the Institutional Review Board of Peking University Third Hospital. Written informed consent for participation was not required for this study in accordance with the national legislation and the institutional requirements.

## Author contributions

SL, ZL, and YW collected and analyzed data and wrote the manuscript. YM, RP, and RQ contributed to data collection. ZZ and HW provided intellectual contributions. HW, ZZ, and WF, supervised the project, discussed data analysis, and reviewed the manuscript. All authors contributed to the article and approved the submitted version.

## Funding

This work was supported by grants from the National Natural Science Foundation of China (Grant No. 62173005), the Peking University Medical Interdisciplinary Research Seed Fund (Grant No. BMU2021MX027, and the National multidisciplinary cooperative diagnosis and treatment capacity building project for major diseases: comprehensive diagnosis and treatment of gastrointestinal tumors.

## Conflict of interest

The authors declare that the research was conducted in the absence of any commercial or financial relationships that could be construed as a potential conflict of interest.

## Publisher’s note

All claims expressed in this article are solely those of the authors and do not necessarily represent those of their affiliated organizations, or those of the publisher, the editors and the reviewers. Any product that may be evaluated in this article, or claim that may be made by its manufacturer, is not guaranteed or endorsed by the publisher.

## Supplementary material

Supplementary Figure 1 Calibration curves of CBP in Training cohort **(A)** and Validation cohort **(E)**. Calibration curve of CP in Training cohort **(B)** and Validation cohort **(F)**. calibration curve of BP in Training cohort **(C)** and Validation cohort **(G)**. calibration curve of and Tan in Training cohort **(D)** and Validation cohort **(H)**.

Supplementary Figure 2 Nomogram of CP **(A)**, BP **(B)** and Tan **(C)**.
