# Supplementary figures and images for: A novel prediction model for pathological complete response based on clinical and blood parameters in locally advanced rectal cancer
Source: Front Oncol. 2022 Nov 23;12:932853. doi: 10.3389/fonc.2022.932853 (PMC9727231; doi:10.3389/fonc.2022.932853)

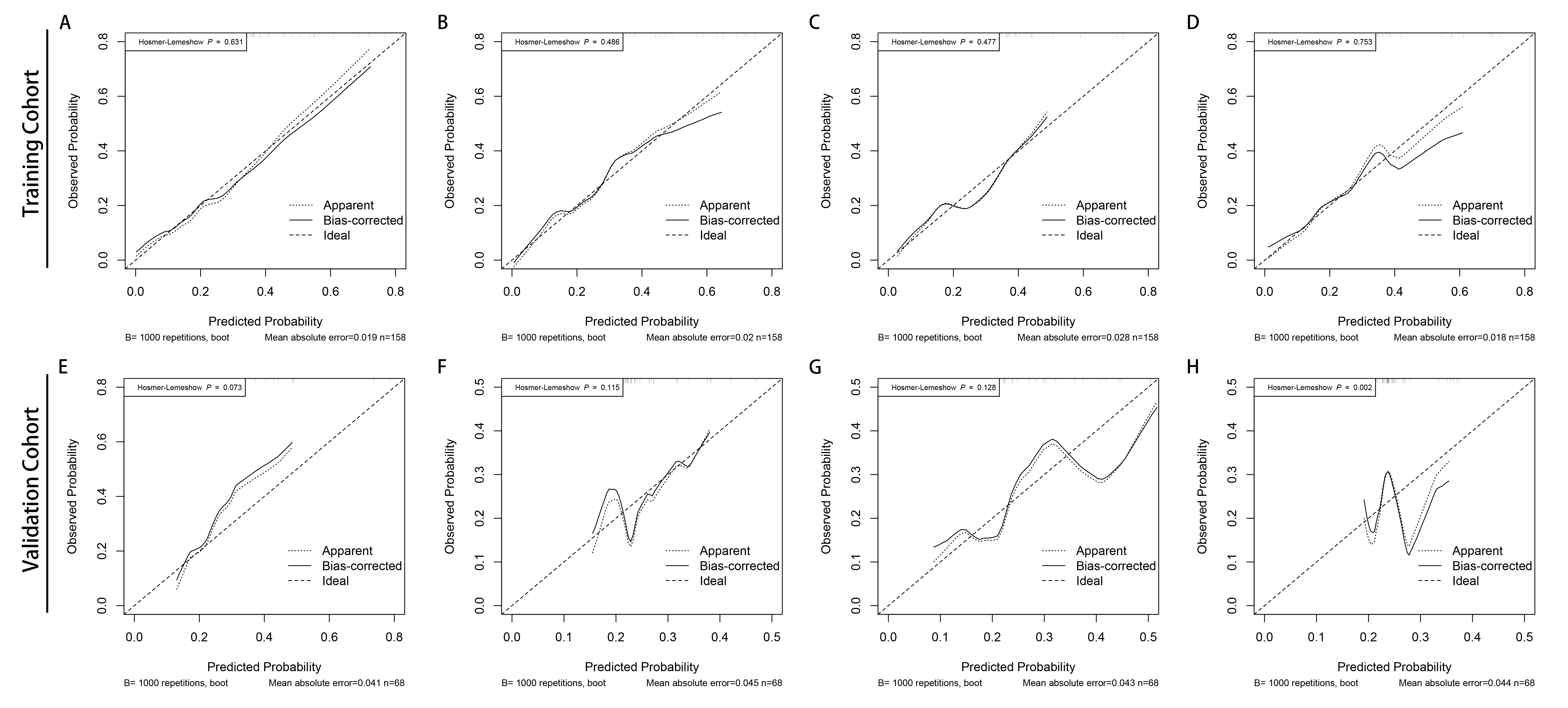

Supplement: Supplementary Figure 1 — Calibration curves of CBP in Training cohort (A) and Validation cohort (E). Calibration curve of CP in Training cohort (B) and Validation cohort (F). calibration curve of BP in Training cohort (C) and Validation cohort (G). calibration curve of and Tan in Training cohort (D) and Validation cohort (H). [file Image_1.tif]

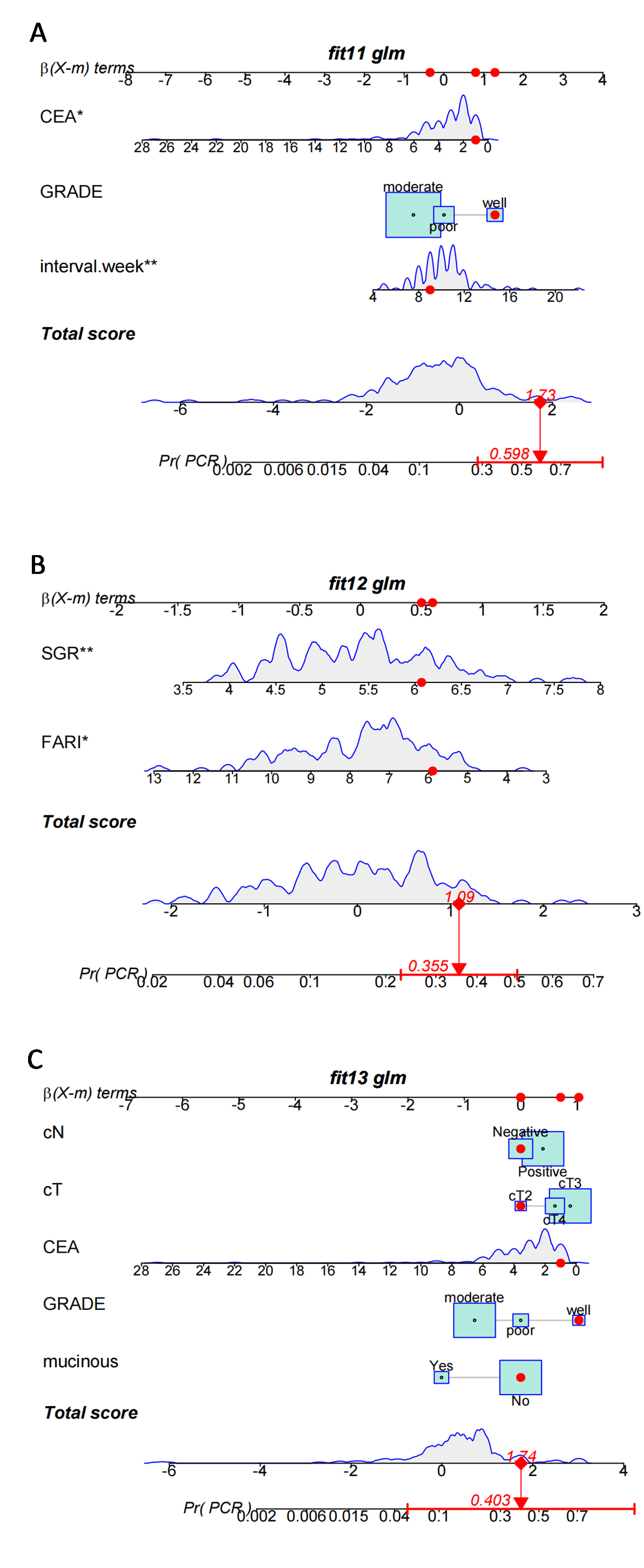

Supplement: Supplementary Figure 2 — Nomogram of CP (A), BP (B) and Tan (C). [file Image_2.tif]
