# Supplementary material for: A novel prediction model for pathological complete response based on clinical and blood parameters in locally advanced rectal cancer
Source: Front Oncol. 2022 Nov 23;12:932853. doi: 10.3389/fonc.2022.932853 (PMC9727231; doi:10.3389/fonc.2022.932853)
Supplement: Supplementary file 3 [file Table_1.docx]

**Supplementary Table 1.** Performance of the four models

|  | **CBP model** | |  | **CP model** | |  | **BP model** | |  | **Tan model** | |
| --- | --- | --- | --- | --- | --- | --- | --- | --- | --- | --- | --- |
| **Metrics** | **Training** | **Validation** |  | **Training** | **Validation** |  | **Training** | **Validation** |  | **Training** | **Validation** |
| AUC | 0.813 | 0.752 |  | 0.762 | 0.589 |  | 0.695 | 0.718 |  | 0.738 | 0.552 |
| Accuracy | 0.816 | 0.779 |  | 0.620 | 0.500 |  | 0.639 | 0.735 |  | 0.677 | 0.529 |
| Sensitivity | 65.5% | 37.5% |  | 79.3% | 62.5% |  | 72.4% | 62.5% |  | 79.3% | 68.8% |
| Specificity | 85.3% | 90.4% |  | 58.1% | 46.2% |  | 62.0% | 76.9% |  | 65.1 | 48.1% |
